# Prenatal omega-3 LCPUFA and symptoms of allergic disease and sensitization throughout early childhood – a longitudinal analysis of long-term follow-up of a randomized controlled trial

**DOI:** 10.1186/s40413-018-0190-7

**Published:** 2018-06-15

**Authors:** K. P. Best, T. R. Sullivan, D. J. Palmer, M. Gold, J. Martin, D. Kennedy, M. Makrides

**Affiliations:** 1 Healthy Mothers, Babies and Children, South Australian Health and Medical Research Institute, Adelaide, South Australia Australia; 2 School of Medicine, University of Adelaide, Adelaide, South Australia Australia; 3 School of Public Health, University of Adelaide, Adelaide, South Australia Australia; 4 School of Medicine, University of Western Australia, Nedlands, Western Australia 6009 Australia; 5 Telethon Kids Institute, University of Western Australia, Subiaco, Western Australia 6008 Australia; 6 Department of Respiratory and Sleep Medicine, Women’s & Children’s Hospital, North Adelaide, South Australia Australia; 7 South Australian Health and Medical Research Institute, 72 King William Road, North Adelaide, South Australia 5006 Australia

**Keywords:** Allergy, Omega-3, Paediatric, Perinatal, Prevention, Pregnancy, Asthma, Allergic disease, Atopy

## Abstract

**Background:**

Randomized controlled trials of prenatal omega (ω-3) long chain polyunsaturated fatty acid (LCPUFA) supplementation are suggestive of some protective effects on allergic sensitization and symptoms of allergic disease in childhood. Due to the nature of the atopic march, investigation of any effects of this prenatal intervention may be most informative when consistently assessed longitudinally during childhood.

**Methods:**

Follow-up of children (*n* = 706) with familial risk of allergy from the Docosahexaenoic Acid to Optimize Mother Infant Outcome (DOMInO) trial. The intervention group received fish oil capsules (900 mg of ω-3 LCPUFA) daily from <21 weeks’ gestation until birth; the control group received vegetable oil capsules without ω-3 LCPUFA. This new longitudinal analysis reports previously unpublished data collected at 1 and 3 years of age. The allergic disease symptom data at 1, 3 and 6 years of age were consistently reported by parents using the "International Study of Asthma and Allergies in Childhood" (ISAAC) questionnaire. Sensitization was determined by skin prick test to age specific, common allergen extracts.

**Results:**

Changes over time in symptoms of allergic disease with sensitization (IgE-mediated) and sensitization did not differ between the groups; interaction *p* = 0.49, *p* = 0.10, respectively. Averaged across the 1, 3 and 6-year assessments, there were no significant effects of prenatal ω-3 LCPUFA supplementation on IgE-mediated allergic disease symptoms (adjusted relative risk 0.88 (95% CI 0.69, 1.12), *p* = 0.29) or sensitization (adjusted relative risk 0.97 (95% CI 0.82, 1.15), *p* = 0.76). Sensitization patterns to common allergens were consistent with the atopic march, with egg sensitization at 1 year strongly associated with house dust mite sensitization at 6 years, (*p* < 0.0001).

**Discussion:**

Although there is some evidence to suggest that maternal supplementation with 900mg ω-3 LCPUFA has a protective effect on early symptoms of allergic disease and sensitization in the offspring, we did not observe any differences in the progression of disease over time in this longitudinal analysis. Further investigation into the dose and timing of ω-3 LCPUFA supplementation, including long-term follow up of children using consistent outcome reporting, is essential to determine whether this intervention may be of benefit as a primary prevention strategy for allergic disease.

**Conclusion:**

Maternal supplementation with 900 mg of ω-3 LCPUFA did not change the progression of IgE-mediated allergic disease symptoms or sensitization throughout childhood from 1 to 6 years.

**Trial registration:**

Australian New Zealand Clinical Trials Registry (ACTRN); DOMInO trial ACTRN12605000569606, early childhood allergy follow up ACTRN12610000735055 and 6-year allergy follow up ACTRN12615000498594.

## Background

The relentless increase in the worldwide prevalence of allergic disease has heightened the need for primary prevention [1]. Although the rise is unlikely to be due to a single factor, there is consensus that the cause may be due to our changing lifestyle, including diet and environment. Diet in industrialized countries has changed dramatically over the last 50 years, particularly in the balance of fats consumed. Optimal health requires a relative balance of omega-3 (ω-3) and omega-6 (ω-6) fatty acids, however studies report that some ‘Western’ diets contain an overabundance of ω-6 fats at the expense of ω-3 fats [2]. The impact of this change may carry heightened importance for the prenatal period, when the developing fetus is reliant solely on the maternal diet.

There is increasing evidence to support modulation of fetal immune development following prenatal ω-3 LCPUFA supplementation [3–5]. Diets high in ω-3 LCPUFA (from fish and fish oils) increase cell membrane docosahexaenoic acid (DHA; 22:6 ω-3) and eicosapentaenoic acid (EPA; 20:5 ω-3), competing with the synthesis of inflammatory arachidonic acid (AA, 20:4, ω − 6), which results in a reduction in prostaglandin E synthesis and inhibition of cytokine and immunoglobulin E (IgE) production associated with allergies [6].

Cohort studies have consistently reported that increased ω-3 LCPUFA consumption during pregnancy may reduce the risk of atopic eczema, asthma and sensitization to house dust mite (HDM) [7], although it is difficult to infer a causal link from these studies given the potential for residual confounding from environmental factors. Published reviews assessing the effect of n–3 LCPUFAs in the primary prevention of allergic disease have been conducted [7–13]. However, not all are systematic and most pool results from varying supplementation periods including pregnancy, pregnancy and lactation or the lactation period only, thus increasing the variability of results and potentially diluting any causal effect of the intervention. Evidence from randomized controlled trials (RCTs) of prenatal supplementation with ω-3 LCPUFA report consistent protective effects on the risk of IgE mediated eczema [14, 15] and sensitization to egg [14–17] at 1 year of age. However, only one of these trials extended follow-up to school age, reporting a reduction in sensitization to HDM only and no effect on parent reported symptoms of IgE-mediated allergic disease at 6 years of age [18]. Two trials from Denmark that have conducted follow up beyond infancy report a protective effect of prenatal ω-3 LCPUFA supplementation (predominantly as EPA) on cumulative wheeze (independent of sensitization) at 3–5 years of age [19] and atopic asthma at 16 and 24 years of age [20, 21].

Longitudinal analyses have long been recognized as an important tool for obtaining high quality evidence about the determinants of development across the lifespan [22]. However, RCT’s of ω-3 LCPUFA supplementation to date, have only reported allergic disease outcomes from distinct follow-up time points, often with inconsistent outcomes which prevent any analysis to investigate change over time. Given the progressive and heterogenous nature of allergic disease, we aim to investigate if prenatal ω-3 LCPUFA supplementation will modify the course of allergen sensitization or allergic disease symptoms over time (from 1 to 6 years of age). Previously published 1 and 3-year of age allergic disease outcomes from our RCT were based on physician examination and diagnosis of allergic disease, [17, 23] whereas the 6-year results were based on parent reported symptoms using the ‘International Study of Asthma and Allergies in Childhood’ (ISAAC) questionnaire [18]. In this new analysis we have combined previously unpublished ISAAC data collected at 1 and 3 years with 6-year data to permit investigation of the effects of prenatal ω-3 LCPUFA supplementation on the progression of consistently parent reported allergic disease symptoms.

## Methods

### Description of participants

**Fig. 1:**
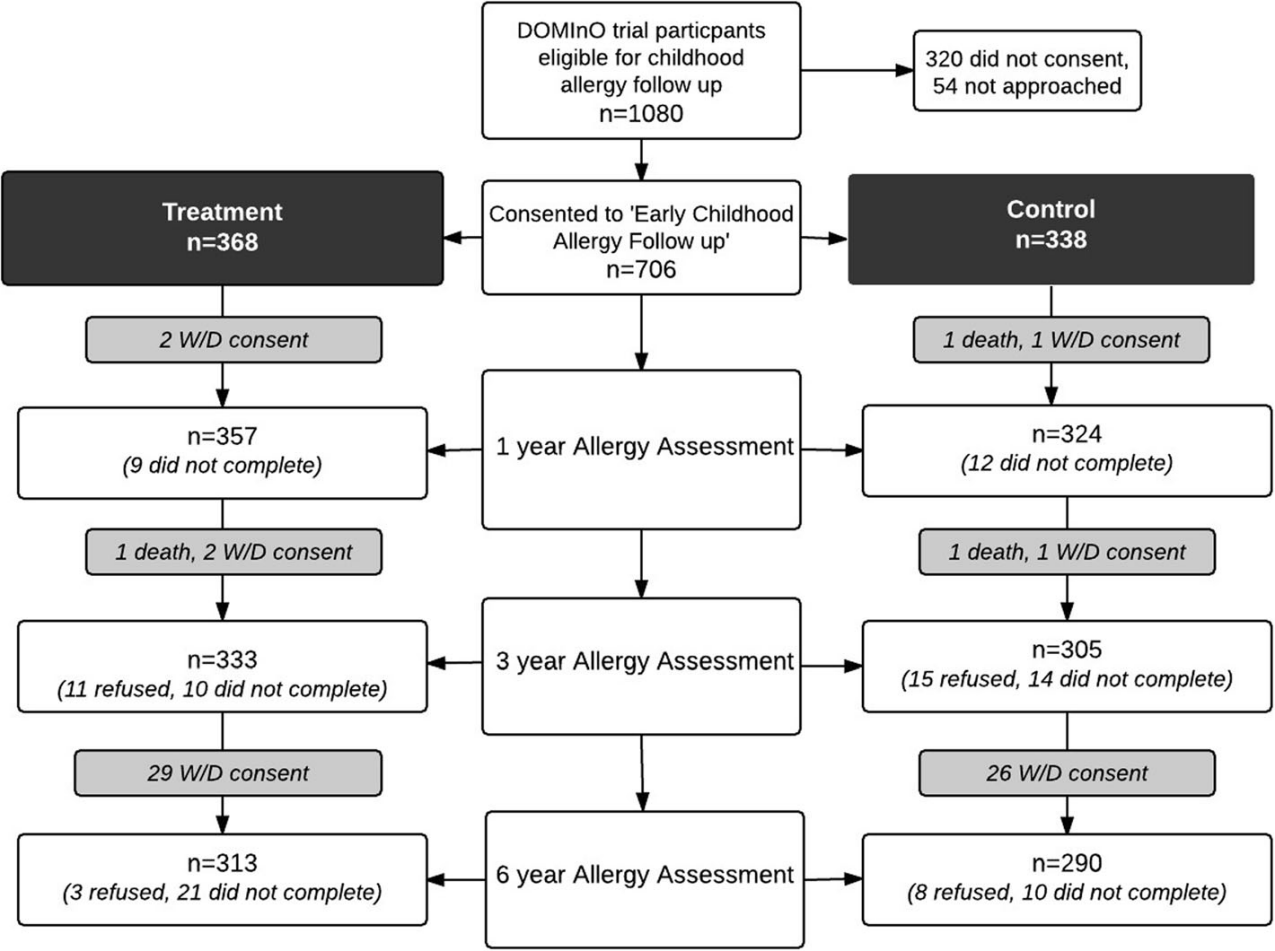
Flow of participants consented to the nested allergy follow-up of the DOMInO trial

This study is a longitudinal synthesis of data obtained at the 1, 3 and 6 year follow up assessment of children at hereditary risk of allergic disease, born to mothers enrolled in the multi-center, double-blind, DOMInO (DHA to Optimize Mother Infant Outcome) RCT [24]. The methods of the DOMInO Trial have been published previously [24]. Briefly, women < 21 weeks’ gestation with a singleton pregnancy were randomly allocated to either treatment or control group through a computer driven telephone randomization service stratified by center and parity (first birth versus subsequent births). Women were asked to consume capsules containing either fish oil concentrate, providing ~ 800 mg/day DHA and 100 mg/day EPA or control capsules containing a blend of three non-genetically modified oils (rapeseed, sunflower, and palm) in equal proportions and designed to match the polyunsaturated, monounsaturated, and saturated fatty acid profile of the average Australian diet [25] (Incromega 500 TG, Croda Chemicals, East Yorkshire, England). The supplementation period commenced at enrolment (< 21 weeks’ gestation) until delivery. All capsules were similar in size, shape, and color, and neither the women nor research staff were aware of the treatment allocated. Women reported their adherence to supplementation at telephone calls at 28 and 36 weeks’ gestation and the concentration of individual LCPUFA in plasma phospholipids from cord blood was assessed as an independent biomarker of compliance. Pregnant women enrolled in the DOMInO Trial from two South Australian Centers (Flinders Medical Center and the Women’s and Children’s Hospital) were invited to participate in an early childhood allergy follow up if their unborn child had a familial history of allergic disease (mother, father, or sibling with a self-reported history of medically diagnosed eczema, asthma, or hay fever). Written informed consent was sought (prior to birth) for the offspring to take part in an allergy assessment at 1 and 3 years of age (*n* = 706). Families of children who consented to the 1 and 3 years of age allergy follow up were contacted again when the child turned 5 years and 9 months and invited to take part in the 6-year of age follow up (*n* = 668), Fig. 1. Results of the early childhood allergy follow up involving physician examination to determine allergic disease outcomes at 1 and 3 years of age, and a subsequent school age follow up study at 6 years of age using ISAAC questionnaire to determine allergic disease symptoms have been previously published [17, 18, 23]. Approval for all allergy follow up was granted by the Human Research Ethics Committees of the Women’s and Children’s Hospital and the Southern Adelaide Health Service, Adelaide, and trials were registered on the Australian New Zealand Clinical Trials Registry (ACTRN); DOMInO trial ACTRN12605000569606, early childhood allergy follow up ACTRN12610000735055 and 6-year allergy follow up ACTRN12615000498594.

### Assessment of allergic disease

Early childhood follow-up appointments at 1 and 3 years of age consisted of physician assessment of allergic disease and interviewer administered parent reported symptom questionnaires using the International study of Asthma and Allergy in Childhood (ISAAC) questionnaire [17, 23]. Additional funding was obtained to conduct a 6 year follow up which did not include a physician assessment. Children attended a clinic appointment and parents were interviewed using ISAAC questionnaires to determine allergic disease symptoms [18]. Allergic disease outcomes included in this longitudinal analysis are based on the parent reported symptoms ascertained via ISAAC questionnaires at 1, 3 and 6 years of age. History of symptoms related to the previous 12-month period and included eczema and wheeze at 1, 3 and 6 years, and rhinitis and rhino-conjunctivitis at 3 and 6 years. Eczema was defined as the history of an itchy rash distributed to the facial, flexural or extensor surface of the skin. Wheeze was defined as a history of wheezing or whistling in the chest. Rhinitis was defined as a history of sneezing or a runny or blocked nose when there have not been symptoms to suggest an upper respiratory tract infection. Rhino-conjunctivitis was defined as a history of sneezing or a runny or blocked nose accompanied by itchy-watery eyes when there have not been symptoms to suggest an upper respiratory tract infection [26]. All ISAAC questionnaires were administered consistently at each time point in accordance with guidelines detailed in the ISAAC user manual [27].

### Assessment of sensitization

Sensitization was assessed at 1, 3 and 6 years by skin prick test (SPT) to common, age specific allergens. One of five experienced research nurses who were blinded to treatment group allocation and specifically trained in pediatric skin prick testing performed the SPTs. SPT’s were performed in a standardized manner and nurses underwent quality assurance reviews every 6 months with one of the investigators. Sensitization was defined as a positive reaction to at least one of the allergen extracts assessed. As per the Australasian Society of Clinical Immunology and Allergy (ASCIA) manual, a test was considered positive if the mean of the horizontal and perpendicular weal diameters was ≥3 mm than that of the negative control site at 15 min [28]. Food allergen extracts were selected according to common foods per age group and aero-allergens were selected based on typical environmental allergens in the geographical area of South Australia, see Table 1. Glycerine and histamine (10 mg/mL) were used as negative and positive controls at each time point. Allergen extracts that were assessed at 2 or more time points were included in the longitudinal analysis.

**Table 1:** Allergen extracts assessed by SPT at 1, 3 and 6 years

1 year	3 years	6 years
Cow’s milk	–	–
Whole hen’s egg	Whole hen’s egg	Whole hen’s egg
Wheat	Wheat	–
Tuna	Tuna	–
Peanut	Peanut	Peanut
–	Cashew	Cashew
–	Sesame	–
Perennial rye grass	Perennial rye grass	Perennial rye grass
Olive tree pollen	Olive tree pollen	Olive tree pollen
*Alternaria tenuis*	*Alternaria tenuis*	*Alternaria tenuis*
Cat	Cat	Cat
–	–	Dog
*D. pteronyssinus*	*D. pteronyssinus*	*D. pteronyssinus*
–	*D. farinae*	*D. farinae*

*Abbreviations: D. pteronyssinus Dermatophagoides pteronyssinus, D. farinae Dermatophagoides farinae*

### Statistical analysis

Longitudinal outcomes were compared between groups using log binomial generalized estimating equations. An independence working correlation matrix was used in each of these models to adjust for the dependence within participants due to repeated measurements over time. In the models the effects of treatment group, time (categorical) and the interaction between treatment group and time were assessed. Adjustment was also made for the stratification variables parity (first birth vs. subsequent birth) and center, as well as the prognostic variables gender and maternal history of allergic disease. Where the interaction term between group and time was not statistically significant, a second model excluding the interaction term was fitted to estimate the effect of treatment over all time points. The effect of treatment, whether at an individual time point or across all time points, was described using relative risks and 95% confidence intervals. All analyses were performed using SAS version 9.3 (SAS Institute Inc., Cary, NC, USA).

## Results

### Sample and participant flow

Enrolment to the nested early childhood allergy follow up began in 2006. A total of 706 offspring were consented between 2006 and 2008 to take part. Of these, 666 (94.3%) infants completed allergy symptom questionnaires and skin prick testing at 1 year of age and 638 (90.3%) at 3 years of age. In 2012, following the exclusion of deaths and withdrawals from the early childhood sample, a total of 668 children remained eligible to participate in the 6-year allergy follow up. Six-year assessments were completed in August 2014 with a total 603/668 (90.0%) allergy symptom questionnaires completed and 485/668 (73.0%) children undergoing SPT to determine sensitization, Fig. 1.

### Baseline characteristics and compliance

The baseline enrolment characteristics of the women and their offspring who were consented to participate in allergy follow-up have been previously described [17] In brief, mean maternal age at trial entry was 29.6 years (SD 5.7 years) and 13.0% of the participating mothers smoked during pregnancy. All unborn children had at least one first-degree relative with a history of medically diagnosed allergic disease; 70% had a history of maternal allergic disease, 54.0% paternal disease and 29.0% allergic disease in both parents. A total of 39.8% were first born children and 47.7% were males. There were no important differences in baseline characteristics at study enrolment between the intervention and control groups.

Compliance with the intervention was high, with 77.0% of mothers in the ω-3 LCPUFA group and 80.0% of mothers in the control group reporting that they had missed zero to three capsules a week (from a total of 21 capsules per week) at 28 weeks’ gestation. DHA concentration in the plasma phospholipids of cord blood from infants in the high-DHA group was greater than control (DHA 3.54 to 13.68% vs control 3.51 to 11.21%; median, 7.2% vs 6.1% total phospholipid fatty acids, *P* < 0.001). Fewer than 2.0% of mothers in each group chose not to take any capsules.

### Allergic disease symptoms

The prevalence of ‘any’ allergic disease symptoms (irrespective of sensitization) increased from 17.5% at 1 year of age to 49.3% at 6 years of age. Allergic disease symptoms with evidence of sensitization were reported less often but with a similar increase in prevalence over time from 5.3% at 1 year of age to 29.0% at 6 years of age. Longitudinal analysis found no evidence that the difference between the ω-3 LCPUFA and control groups in the risk of ‘any’ IgE mediated allergic disease or ‘individual IgE mediated allergic disease symptoms (eczema, rhinitis, rhino-conjunctivitis or wheeze)’ changed over time (group by time interaction *p*-values > 0.05). After excluding the group by time interaction effect from the models, there were no significant differences between the prenatal ω-3 LCPUFA supplementation and control groups in the risk of ‘any’ IgE-mediated allergic disease or individual IgE-mediated allergic disease symptoms (eczema, rhinitis, rhino-conjunctivitis, and wheeze) with sensitization across all years (group *p*-values > 0.05), Table 2. Although a difference between groups was observed at 1 year of age (aRR 0.51, 95% CI 0.28 to 0.92, *P* = 0.03), the effect did not persist at 3 and 6 years, such that overall the longitudinal analysis did not provide any evidence of an effect of ω-3 LCPUFA supplementation.

**Table 2:** Longitudinal analysis of allergic disease symptoms with sensitization at 1, 3 and 6 years of age

Outcome	Year	ω-3 LCPUFA^a^ n (%)	Control^a^ n (%)	RR (95% CI)	*P*-value	Interaction *p*-value	Adjusted RR^b^ (95% CI)	Adjusted *p*-value	Interaction *p*-value^b^
Any allergic disease symptoms with sensitization	1	15/363 (4.13%)	22/330 (6.67%)	0.62 (0.33, 1.17)	0.14	0.51	0.63 (0.33, 1.19)	0.16	0.49
	3	58/313 (18.53%)	61/291 (20.96%)	0.88 (0.64, 1.22)	0.45		0.89 (0.65, 1.23)	0.48	
	6	76/280 (27.14%)	82/263 (31.18%)	0.87 (0.67, 1.13)	0.30		0.92 (0.71, 1.19)	0.54	
	*All years*			*0.85 (0.66, 1.08)*	*0.18*		*0.88 (0.69, 1.12)*	*0.29*	
Rhinitis symptoms with sensitization	3	25/327 (7.65%)	28/301 (9.30%)	0.82 (0.49, 1.38)	0.46	0.90	0.83 (0.50, 1.39)	0.48	0.87
	6	55/298 (18.46%)	60/276 (21.74%)	0.85 (0.61, 1.18)	0.33		0.87 (0.63, 1.20)	0.39	
	*All years*			*0.84 (0.62, 1.14)*	*0.27*		*0.86 (0.63, 1.16)*	*0.32*	
Rhino-conjunctivitis symptoms with sensitization	3	12/331 (3.63%)	20/304 (6.58%)	0.55 (0.27, 1.11)	0.09	0.16	0.55 (0.28, 1.11)	0.09	0.16
	6	34/304 (11.18%)	33/281 (11.74%)	0.95 (0.61, 1.49)	0.83		0.96 (0.61, 1.51)	0.87	
	*All years*			*0.81 (0.54, 1.21)*	*0.30*		*0.81 (0.55, 1.21)*	*0.31*	
Wheeze symptoms with sensitization	1	16/357 (4.48%)	29/329 (8.81%)	0.51 (0.28, 0.92)	0.03	0.13	0.52 (0.29, 0.94)	0.03	0.13
	3	36/317 (11.36%)	35/295 (11.86%)	0.96 (0.62, 1.48)	0.84		0.96 (0.63, 1.49)	0.87	
	6	38/290 (13.10%)	38/276 (13.77%)	0.95 (0.63, 1.45)	0.82		0.99 (0.65, 1.51)	0.97	
	*All years*			*0.83 (0.60, 1.15)*	*0.26*		*0.85 (0.62, 1.17)*	*0.33*	
Eczema symptoms with sensitization	1	15/363 (4.13%)	22/330 (6.67%)	0.62 (0.33, 1.17)	0.14	0.68	0.63 (0.34, 1.18)	0.15	0.64
	3	34/324 (10.49%)	41/301 (13.62%)	0.77 (0.50, 1.18)	0.23		0.78 (0.51, 1.19)	0.25	
	6	24/303 (7.92%)	26/277 (9.39%)	0.84 (0.50, 1.43)	0.53		0.88 (0.52, 1.50)	0.65	
	*All years*			*0.76 (0.52, 1.11)*	*0.15*		*0.77 (0.53, 1.13)*	*0.19*	

For ω-3 LCPUFA and control groups, data are number of subjects (percentage)

Rhinitis & Rhino-conjunctivitis symptoms not assessed at 1 year

Abbreviations: *CI* confidence interval, *ω-3 LCPUFA* long chain polyunsaturated fatty acid, *RR* relative risk

^a b^Adjusted for enrolling centre, parity, child sex and maternal history of allergic disease

### Analysis of sensitization to individual allergen extracts

**Fig. 2:**
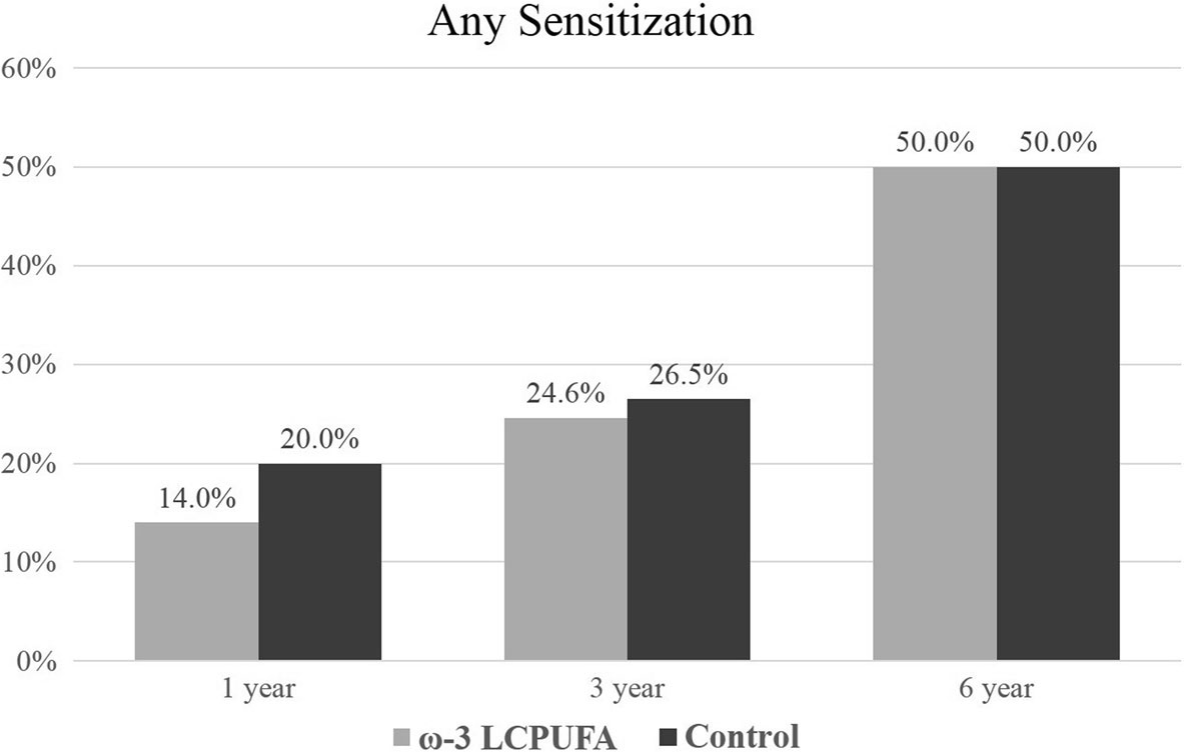
Sensitization to one or more allergen extracts at 1, 3 and 6 years

**Fig. 3:**
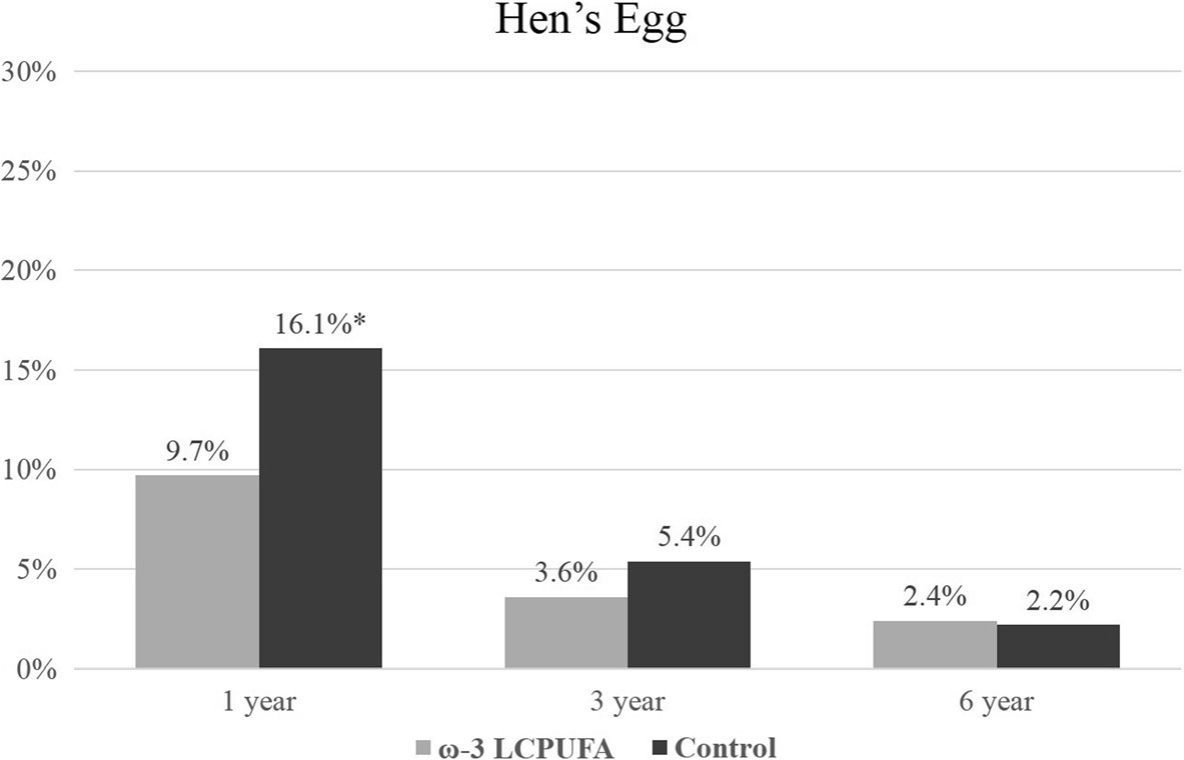
Sensitization to hen’s egg at 1, 3 and 6 years (*denotes *p* = < 0.05)

**Fig. 4:**
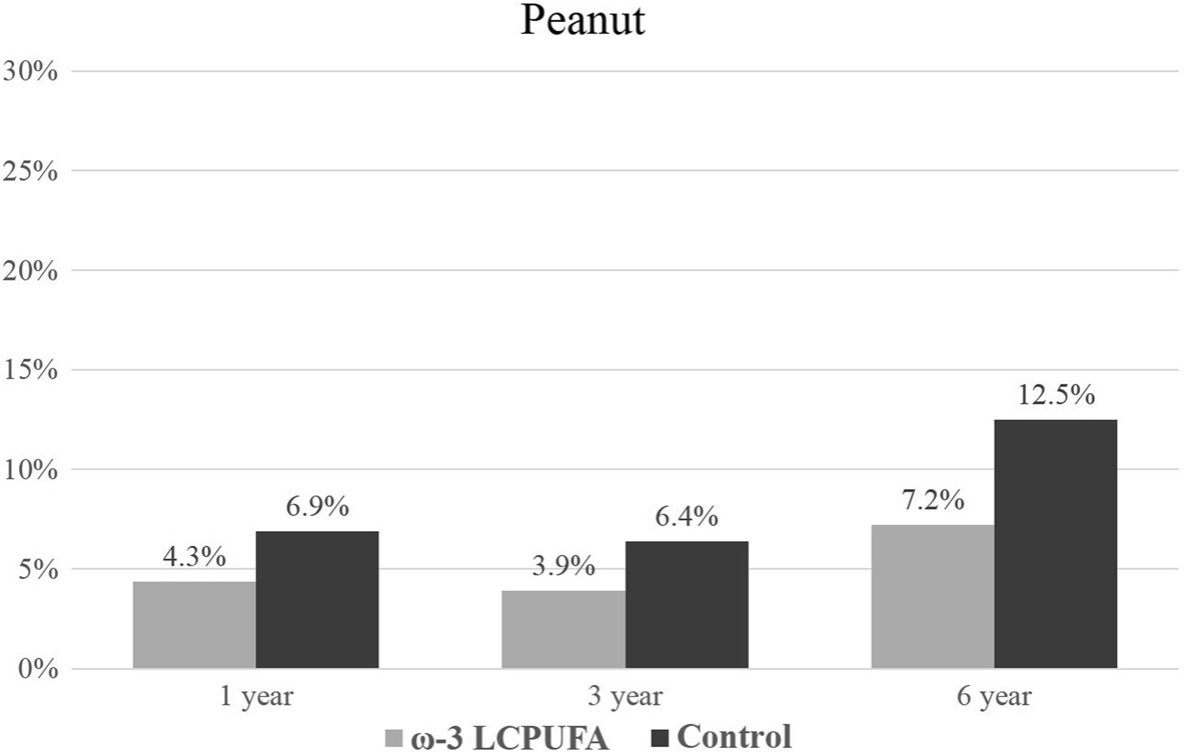
Sensitization to peanut 1, 3 and 6 years

**Fig. 5:**
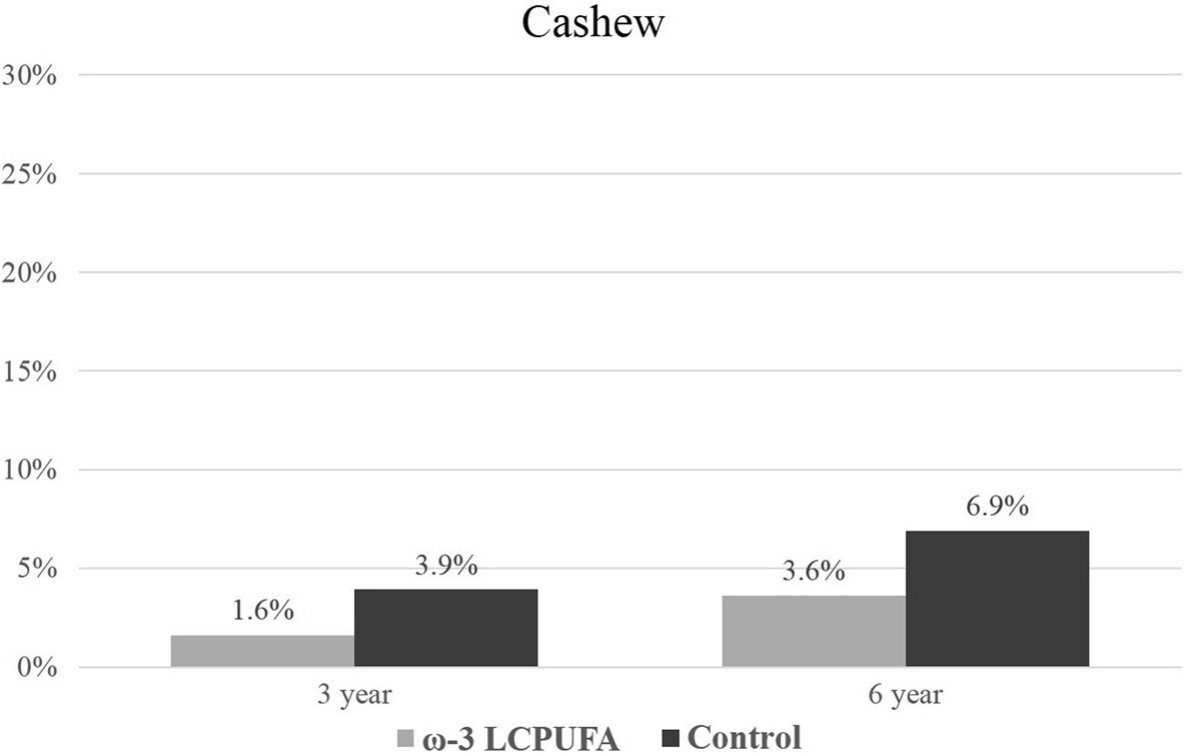
Sensitization to cashew at 3 and 6 years

**Fig. 6:**
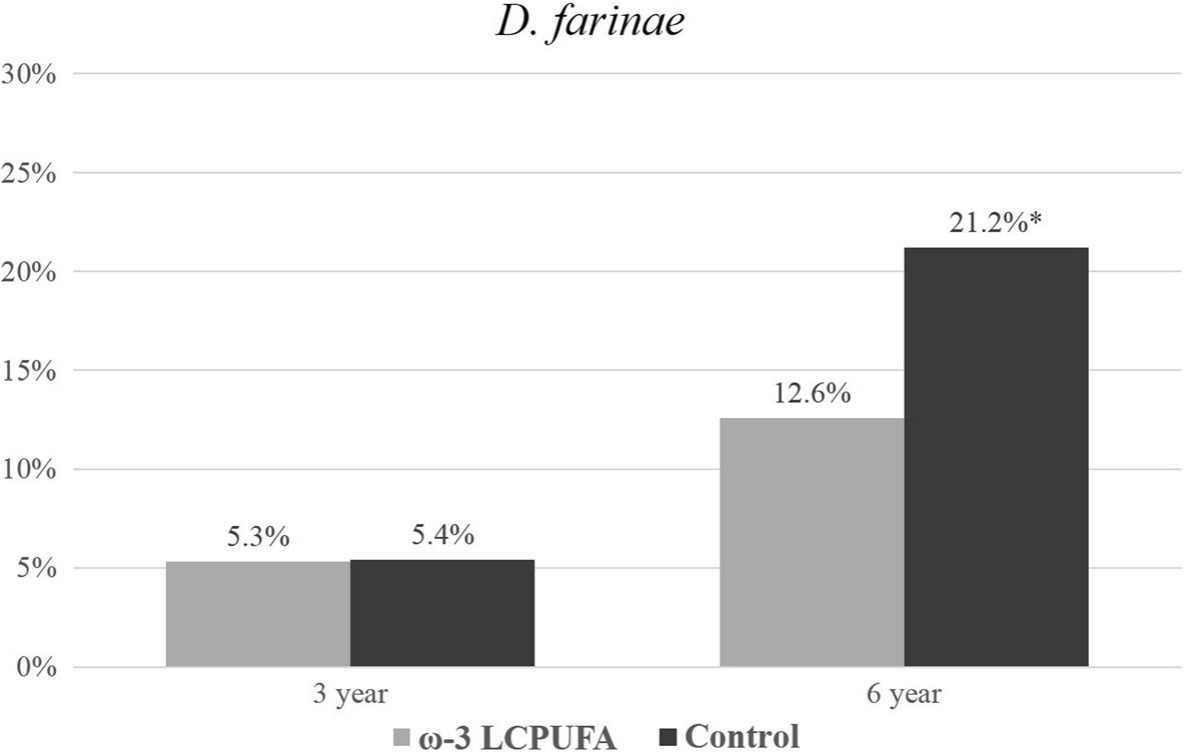
Sensitization to D. farinae at 3 and 6 years (*denotes *p* = < 0.05)

**Fig. 7:**
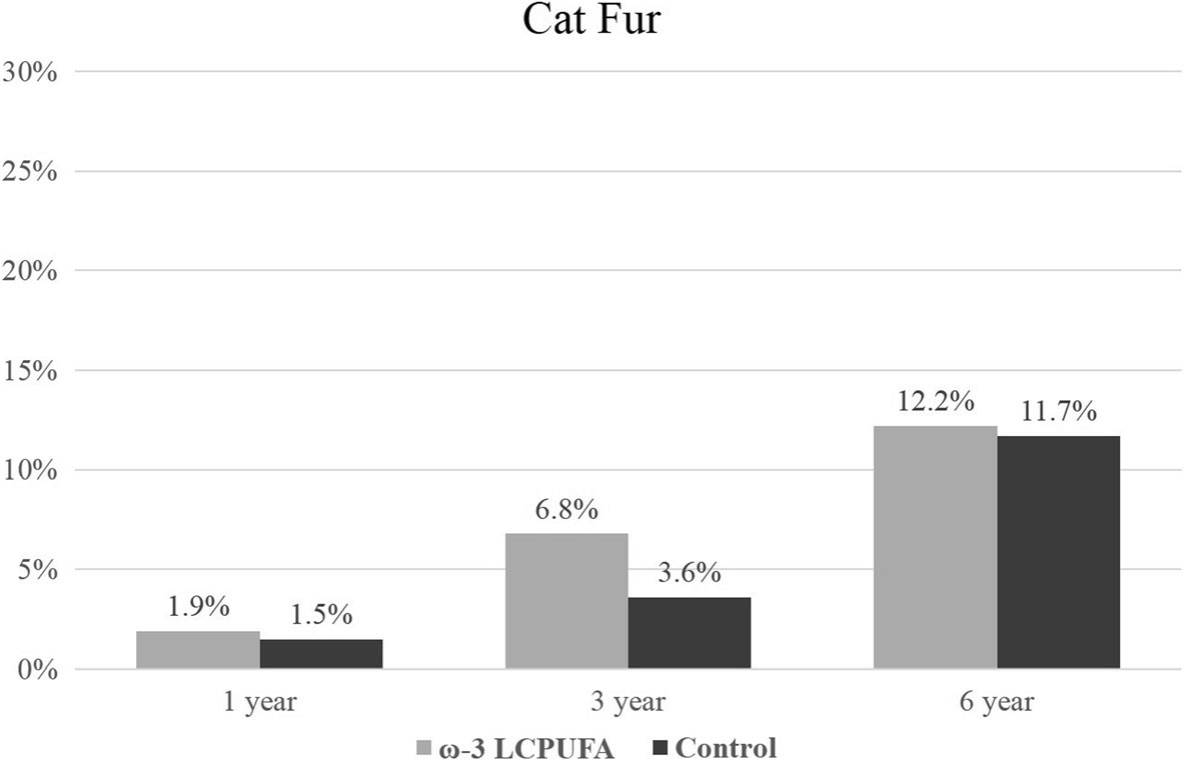
Sensitization to cat at 1, 3 and 6 years

The prevalence of ‘any sensitization’ (≥1 positive SPT reaction to any allergen extract) increased with the age of the child from 17.0% at 1 year of age to 50.0% at 6 years of age. Sensitization outcomes were included in the longitudinal analysis if the allergen extract was assessed at two or more time-points. Sensitization results for individual allergen extracts at 1, 3 and 6 years are presented in Figs. 2, 3, 4, 5, 6 and 7. Longitudinal analysis found little evidence that the effect of treatment on ‘any sensitization’ or sensitization to individual allergen extracts changed over time (group by time interaction *P*-values > 0.05). After excluding interaction effects, there were no significant differences between groups in the risk of ‘any sensitization’ or ‘sensitization to individual allergen extracts’ across all years (group *p*-values > 0.05). However, the direction of effect was similar for many of the extracts across all years. The relative risk associated with ω-3 LCPUFA supplementation ranged between 0.50 and 0.70 for hen’s egg, peanut, cashew and D. *farinae*, Table 3.

**Table 3:** Longitudinal analysis of sensitization to allergen extracts at 1, 3 and 6 years of age

Outcome	Year	ω-3 LCPUFA^a^ n (%)	Control^a^ n (%)	RR (95% CI)	*P*-value	Interaction *p*-value	Adjusted RR^b^ (95% CI)	Adjusted *p*-value	Interaction *p*-value^b^
Any Sensitization	1	50/349 (14.33%)	63/317 (19.87%)	0.72 (0.51, 1.01)	0.06	0.16	0.73 (0.52, 1.02)	0.07	0.10
	3	77/303 (25.41%)	74/279 (26.52%)	0.96 (0.73, 1.26)	0.76		0.97 (0.74, 1.27)	0.83	
	6	124/248 (50.00%)	116/232 (50.00%)	1.00 (0.84, 1.20)	> 0.99		1.05 (0.88, 1.25)	0.56	
	*All years*			*0.94 (0.79, 1.12)*	*0.47*		*0.97 (0.82, 1.15)*	*0.76*	
Egg sensitization‡	1	34/349 (9.74%)	51/317 (16.09%)	0.61 (0.40, 0.91)	0.02	0.55	0.62 (0.42, 0.93)	0.02	0.54
	3	11/307 (3.58%)	15/280 (5.36%)	0.67 (0.31, 1.43)	0.30		0.68 (0.32, 1.44)	0.31	
	6	6/248 (2.42%)	5/232 (2.16%)	1.12 (0.35, 3.63)	0.85		1.16 (0.36, 3.73)	0.81	
	*All years*			*0.65 (0.42, 1.01)*	*0.05*		*0.67 (0.43, 1.02)*	*0.06*	
Peanut sensitization	1	15/349 (4.30%)	22/317 (6.94%)	0.62 (0.33, 1.17)	0.14	0.98	0.65 (0.34, 1.22)	0.18	0.98
	3	12/307 (3.91%)	18/280 (6.43%)	0.61 (0.30, 1.24)	0.17		0.63 (0.31, 1.27)	0.20	
	6	18/250 (7.20%)	29/232 (12.50%)	0.58 (0.33, 1.01)	0.05		0.61 (0.35, 1.06)	0.08	
	*All years*			*0.60 (0.37, 0.98)*	*0.04*		*0.63 (0.39, 1.02)*	*0.06*	
Cat sensitization	1	7/349 (2.01%)	5/317 (1.58%)	1.27 (0.41, 3.97)	0.68	0.11	1.30 (0.42, 4.00)	0.65	0.12
	3	24/303 (7.92%)	11/277 (3.97%)	1.99 (1.00, 4.00)	0.05		2.02 (1.02, 4.02)	0.04	
	6	30/247 (12.15%)	27/231 (11.69%)	1.04 (0.64, 1.69)	0.88		1.07 (0.66, 1.74)	0.79	
	*All years*			*1.30 (0.80, 2.13)*	*0.29*		*1.34 (0.83, 2.18)*	*0.23*	
Cashew sensitization	3	5/307 (1.63%)	11/280 (3.93%)	0.41 (0.15, 1.18)	0.10	0.56	0.43 (0.15, 1.20)	0.11	0.50
	6	9/249 (3.61%)	16/231 (6.93%)	0.52 (0.24, 1.16)	0.11		0.55 (0.25, 1.25)	0.15	
	*All years*			*0.48 (0.21, 1.08)*	*0.08*		*0.50 (0.22, 1.14)*	*0.10*	
*D. Farinae* sensitization	3	16/303 (5.28%)	15/277 (5.42%)	0.98 (0.49, 1.94)	0.94	0.15	0.98 (0.50, 1.94)	0.96	0.18
	6	31/246 (12.60%)	49/231 (21.21%)	0.59 (0.39, 0.90)	0.01		0.62 (0.41, 0.93)	0.02	
	*All years*			*0.68 (0.46, 1.00)*	*0.05*		*0.70 (0.47, 1.03)*	*0.07*	

For ω-3 LCPUFA and control groups, data are number of subjects (percentage)

Longitudinal analysis not performed for sensitization to ryegrass, olive, D. pteronyssinus and alternaria tenuis due to insufficient cases at 1/3 years

Abbreviations: *CI* confidence interval, *ω-3 LCPUFA* long chain polyunsaturated fatty acid, *RR* relative risk

^a b^Adjusted for enrolling centre, parity, child sex and maternal history of allergic disease

### Hen’s egg and HDM sensitization associations

Although there was no evidence for a change in risk across all years, ω-3 LCPUFA supplementation was observed to decrease the risk of hen’s egg sensitization at 1 year and HDM sensitization at 6 years, Table 3. Based on these results, in exploratory analyses we investigated the relationship between these two sensitization measures. Overall, the risk of HDM sensitization at 6 years of age was 40.3% in children with egg sensitization at 1 year of age compared to 13.2% in children without egg sensitization (*P* < 0.0001), with the association more pronounced in the ω-3 LCPUFA group (increase from 9.7 to 37.5%) than in the control group (17.3 to 42.1%). Given the strong association between sensitization outcomes, a mediation analysis was undertaken to test whether the effect of treatment on HDM sensitization at 6 years of age was mediated through earlier effects on egg sensitization. Since adjustment for egg sensitization at 1 year had little impact on the estimated effect of treatment on HDM sensitization at 6 years (relative risk changing from 0.62 without adjustment, to 0.69 with adjustment), it seems that most of the effect of treatment on HDM sensitization was unrelated to earlier effects on egg sensitization.

## Discussion

This longitudinal analysis of our nested allergy cohort from the DOMInO Trial [24] was designed to determine the effect of prenatal ω-3 LCPUFA supplementation on parent reported allergic disease symptoms and sensitization over time. Our results show that a maternal dose of 900 mg of ω-3 LCPUFA per day during pregnancy had no effect on symptoms of allergic disease or sensitization over time.

Long-term follow-up of perinatal interventions is critical to evaluate effects which may manifest themselves only after several years [29]. This is particularly important for prenatal interventions aimed at primary prevention of allergic disease in which the timing of onset, the nature of clinical symptoms and sensitization to specific allergens is known to be heterogeneous [30–33]. Longitudinal analysis of multiple assessment throughout long-term follow-up is the optimal method to determine effects over time. To ensure consistency in outcome reporting we combined previously unpublished parent reported symptoms of allergic disease (collected using ISAAC questionnaire) at 1 and 3 years of age with 6-year ISAAC data.

The progression in the prevalence of parent reported allergic disease symptoms was consistent with the atopic march and increased from 17.5% at 1 year of age to 49.3% at 6 years of age. IgE mediated allergic disease symptoms (symptoms and sensitization to ≥1 allergen extract) were reported less often with 5.3% at 1 year of age increasing to 29.0% at 6 years of age, longitudinal analysis found no significant differences between the ω-3 LCPUFA and control groups over time. Our previous work has reported a significant reduction in sensitization to egg at 1 year of age [17] and HDM at 6 years of age [18] following maternal ω-3 LCPUFA supplementation, however our longitudinal analysis did not show any age dependent effects. Our results strongly support known associations between early sensitization to food protein and later sensitization to aero-allergens [34–36]. Prenatal ω-3 LCPUFA supplementation was observed to significantly decrease the risk of hen’s egg sensitization at 1 year of age and HDM sensitization at 6 years of age with the association more pronounced in the ω-3 LCPUFA group. There is growing evidence that early-life sensitization to hen’s egg and sensitization to HDM predict asthma and rhino-conjunctivitis in childhood [37] adolescence [38] and adulthood [30]. We anticipated that the reduction in HDM sensitization would be a cascade effect of the effects of the intervention on egg sensitization at 1-year of age, however, mediation analysis to investigate sensitization associations, showed that most of the effect of treatment on HDM sensitization was unrelated to earlier effects on egg sensitization (relative risk changing from 0.62 without adjustment, to 0.69 with adjustment).

The majority of RCTs designed to investigate the effect of prenatal ω-3 LCPUFA supplementation on childhood allergy have been driven by the plausible hypothesis that diets high in ω-3 LCPUFAs may modulate the development of immunoglobulin E (IgE) associated allergic disease [14–18, 23]. Whilst there is a level of consistency with reports of protective effects of ω-3 LCPUFA on some sensitization outcomes, a lack of congruence regarding effects on allergic disease symptoms persist. Disparity in results between trials may be attributed to several factors including, but not limited to; timing and dose of the intervention, type of intervention (predominantly DHA vs. EPA), inconsistent outcome reporting, the absence of long term follow-up of outcomes and methodological issues. In regard to timing of supplementation, post-natal supplementation alone (either directly to the infant or via breastmilk) has not proved beneficial in the reduction of allergic disease [13, 39]. Only one trial has continued prenatal ω-3 LCPUFA supplementation throughout the post-natal period [14]. Whilst sample size of this trial was small and offspring follow up was ceased at 2 years of age, results suggest that continued supplementation of breastfeeding mothers to compensate for the natural decline in maternal plasma ω-3 LCPUFAs, may provide additional benefit to the development and maturation of the infant immune system. Compared with our study, other studies have used much higher doses of ω-3 LCPUFA that have ranged from 2700 mg/day [14, 19–21] to 3700 mg/day [16]. With benefit of the increasing evidence in this field now available, we recognize that our dose of ω-3 LCPUFA may not have been adequate and it may be, that higher doses are needed to see a reduction in clinical outcomes of allergic disease. However, we are also cognizant of potential adverse effects of ω-3 LCPUFA supplementation as seen by the increased rate of obstetric interventions in the arm of the DOMInO Trial due to an increase in length of gestation in the intervention group [24].

To the best of our knowledge, this is the first longitudinal analysis of sensitization and parent reported allergic disease symptoms in the offspring following maternal ω-3 LCPUFA supplementation. The major strength of our work is the use of data from the DOMInO Trial which was primarily designed to investigate post-natal depression in women and neurodevelopment in children [24]. Long-term follow up of children from this well conducted RCT with excellent follow up rates and a low risk of bias has enabled a longitudinal analysis of allergic disease outcomes that has not been possible in previous trials. Our consistently collected outcome measures have enabled combination of data from multiple time points into specific analyses that investigate change over time.

A limitation of this analysis may be the use of the ISAAC questionnaire for the diagnosis of allergic disease symptoms, particularly in infancy given that it has only been validated for 6–7 and 13–14 year old children [40]. There were some discrepancies between overall prevalence of allergic disease diagnosis determined by parental reported symptoms from ISAAC questionnaires at 1 and 3 years of age and physician assessment. At 1 year of age, eczema with sensitization was reported less often using ISAAC questionnaires compared to physician diagnosis (3.5% vs. 9.2%) whilst at 3 years of age, wheeze with sensitization was more frequently reported by ISAAC questionnaires than physician diagnosed asthma (11.6% vs. 1.6%). This difference in outcome measures highlights the complexity of using wheeze a proxy for asthma diagnosis, particularly in children under pre-school age (< 6 years) when the many wheezing conditions of childhood are prevalent. This disparity in outcome measures further highlight the difficulties surrounding consistent methods for allergic disease symptom definition and the importance of standardization of core outcome reporting.

## Conclusion

Although there is some evidence to suggest that maternal supplementation with 900 mg ω-3 LCPUFA has a protective effect on early symptoms of allergic disease and sensitization, we did not observe any differences in the progression of disease over time in this longitudinal analysis. Further investigation into patterns of sensitization, classification of atopy phenotypes and harmonization of clinical symptom recording are essential to progress this field. Long term follow-up of offspring (in adolescence and adulthood) following prenatal interventions are essential to determine true effects on the trajectory of disease and to confirm whether prenatal ω-3 LCPUFA supplementation may be of benefit as a primary prevention strategy.

## Abbreviations

AA: Arachidonic acid
ACTRN: Australian New Zealand Clinical Trials Registry
aRR: Adjusted relative risk
CI: Confidence interval
D. *farinae*: *Dermatophagoides farinae*
*D. pteronyssinus*: *Dermatophagoides pteronyssinus*
DHA: Docosahexaenoic acid
DOMInO: DHA to Optimize Mother Infant Outcome
EPA: Eicosapentaenoic acid
HDM: House dust mite
IgE: Immunoglobulin E
ISAAC: International Study of Asthma and Allergies in Childhood
LCPUFA: Long-chain polyunsaturated fatty acids
omega-3: ω − 3
omega-6: ω − 6
RCT: Randomized controlled trial
SPT: Skin prick test
